# Supplementary figures and images for: Peptide nucleic acids can form hairpins and bind RNA-binding proteins
Source: PLoS One. 2024 Sep 16;19(9):e0310565. doi: 10.1371/journal.pone.0310565 (PMC11404819; doi:10.1371/journal.pone.0310565)

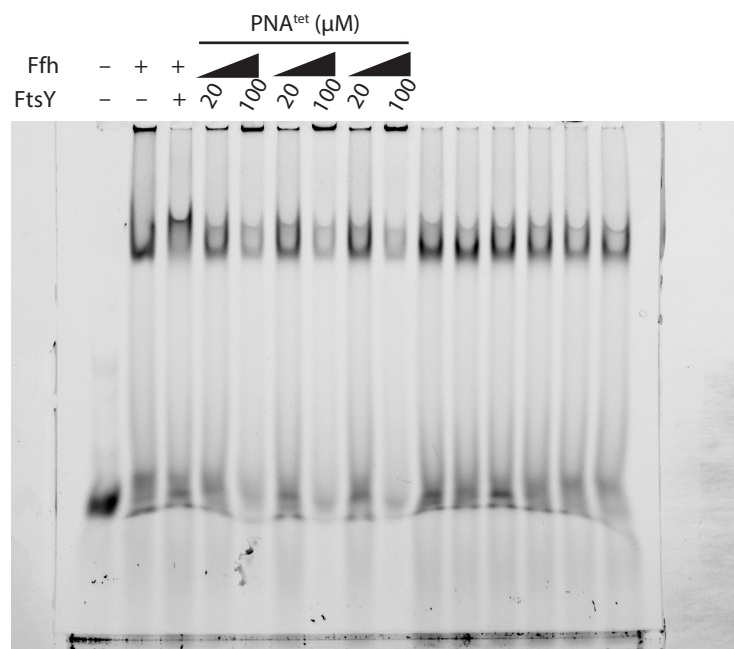

Fig 2D and S Fig 5 right

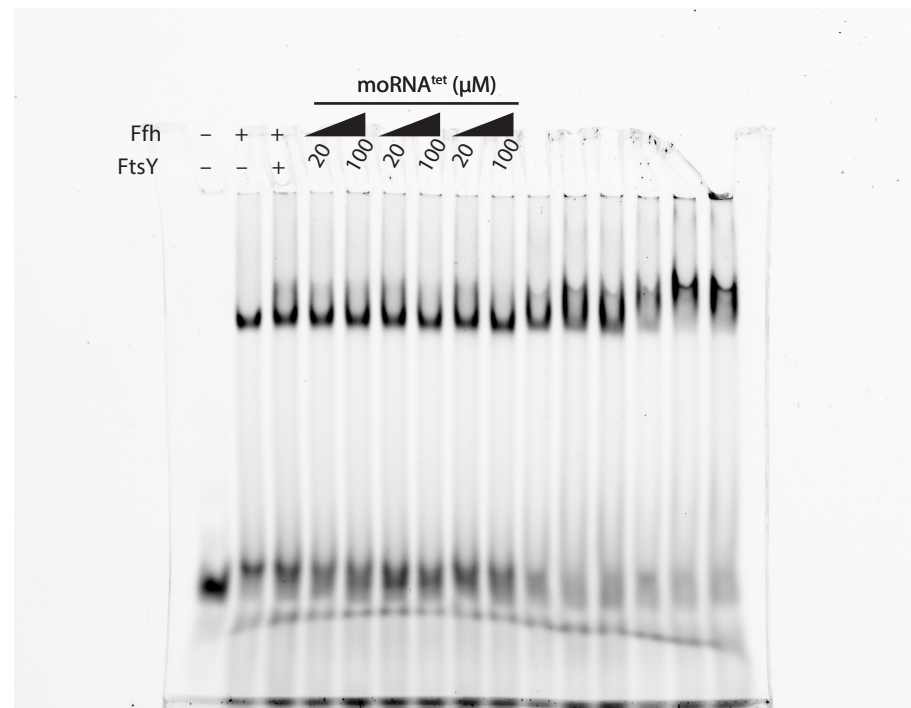

S Fig 5 left

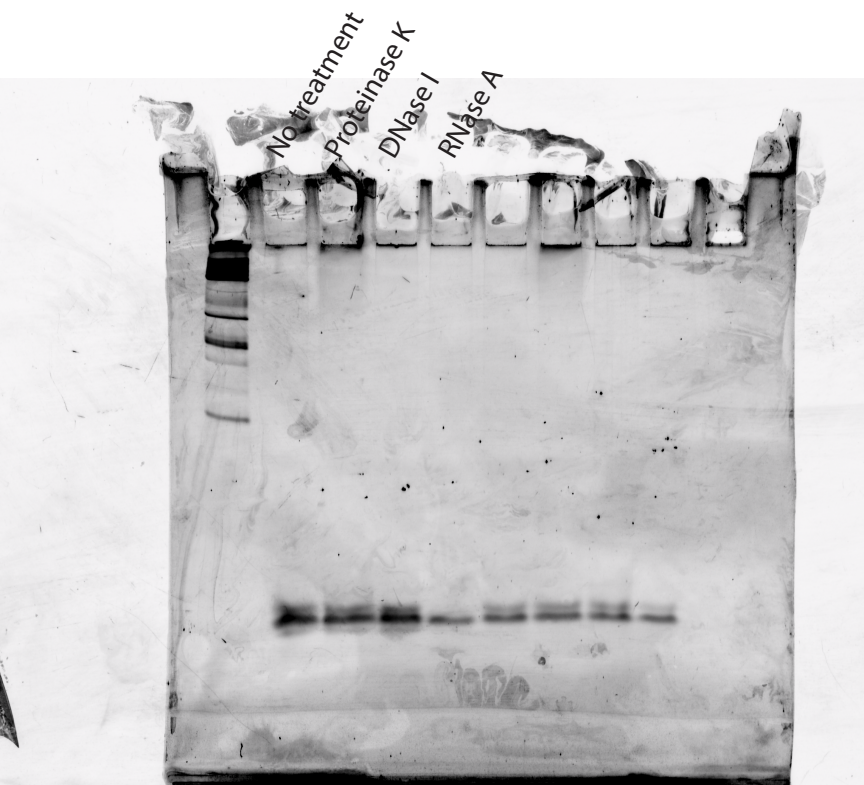

S Fig 6B left unmodified RNA<sup>tet</sup>

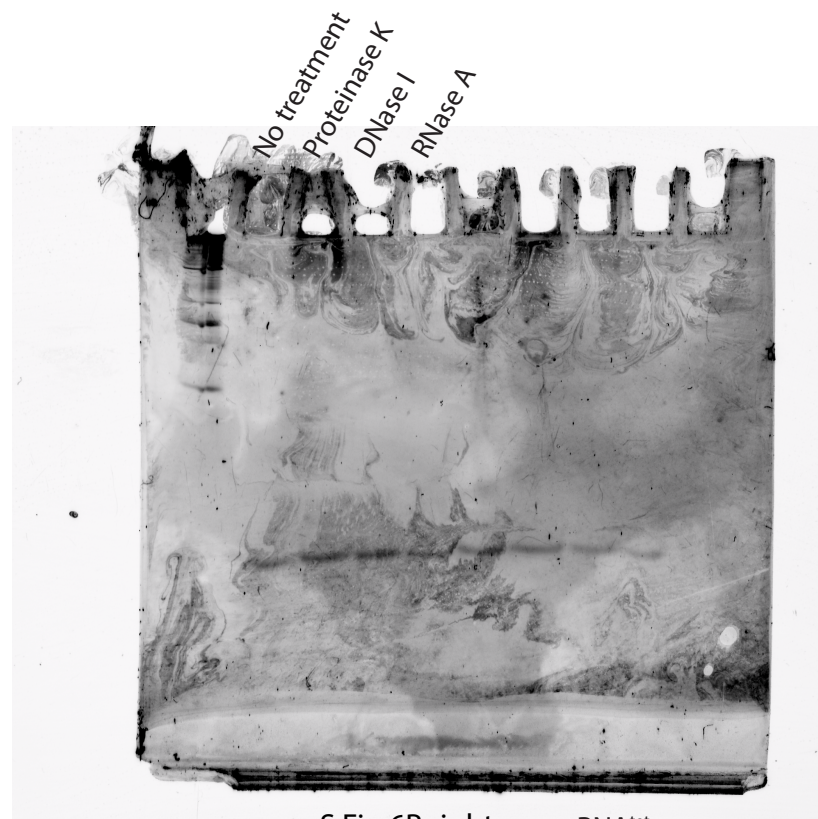

S Fig 6B right moRNA<sup>tet</sup>

Supplement: S1 Raw images — (PDF) [file pone.0310565.s001.pdf]

210 nm

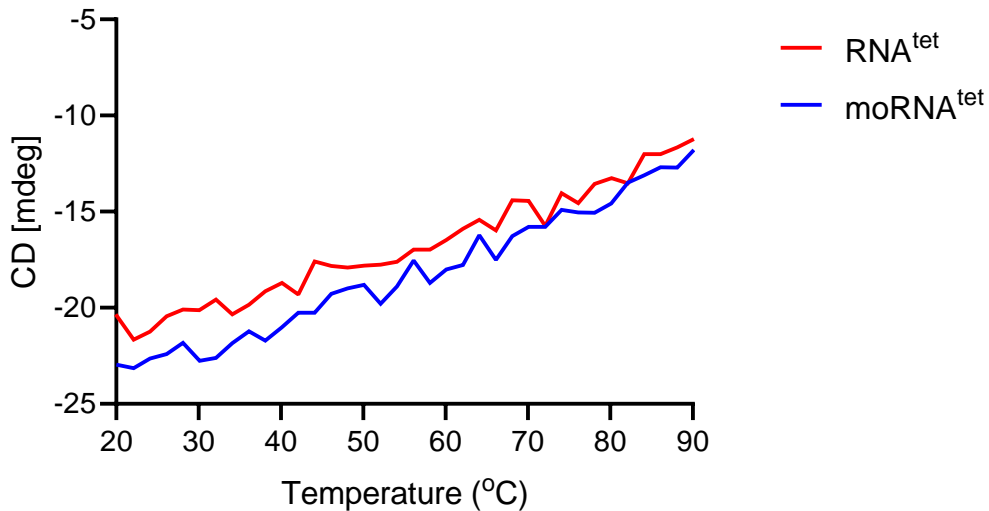

Supplement: S2 File — (ZIP) [file pone.0310565.s003.zip › Raw files/CD/210 nm melt.pdf]

265 nm

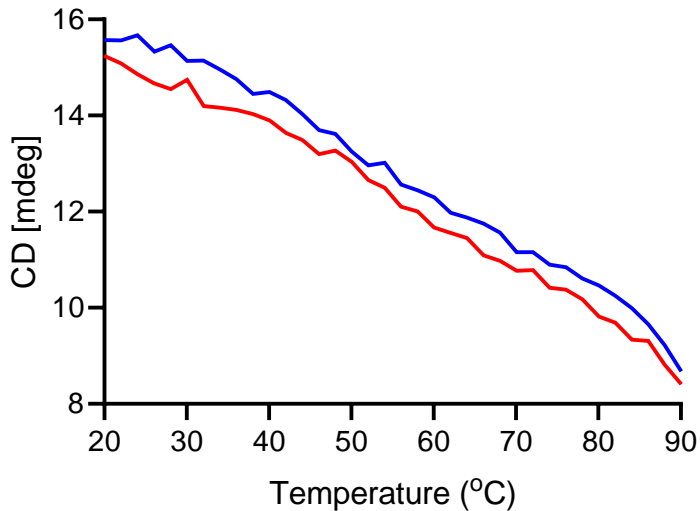

Supplement: S2 File — (ZIP) [file pone.0310565.s003.zip › Raw files/CD/265 nm melt.pdf]

# moRNA<sup>tet</sup>

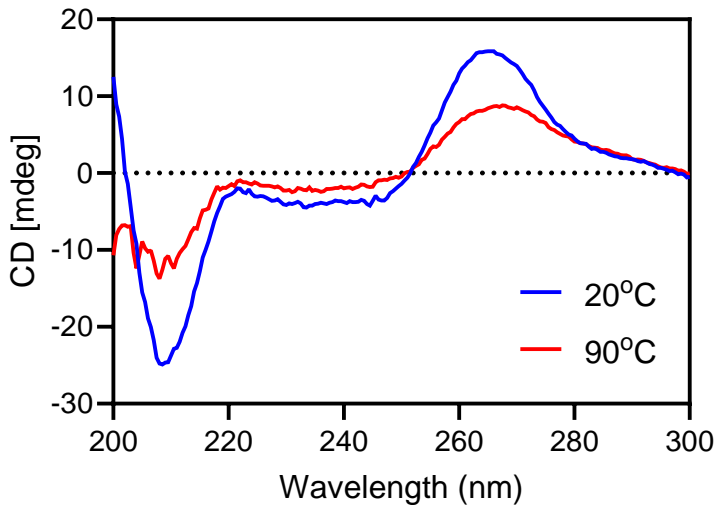

Supplement: S2 File — (ZIP) [file pone.0310565.s003.zip › Raw files/CD/moRNAtet CD Spectrum.pdf]

RNA<sup>tet</sup>

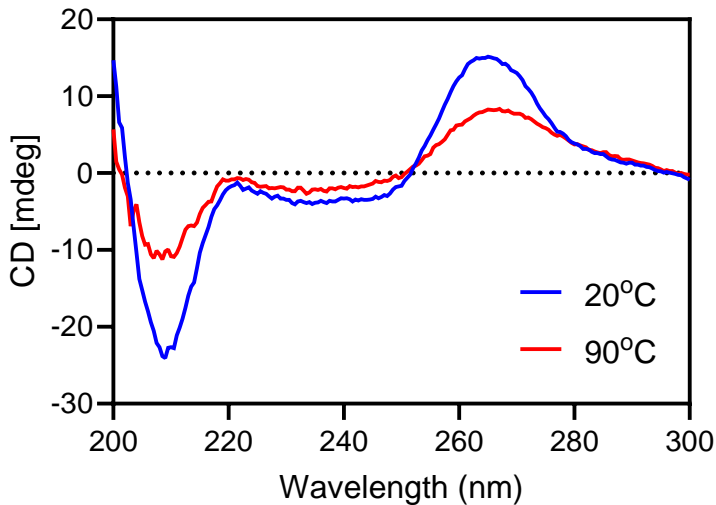

Supplement: S2 File — (ZIP) [file pone.0310565.s003.zip › Raw files/CD/RNAtet CD Spectrum.pdf]

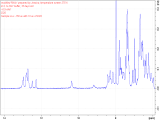

Supplement: S2 File — (ZIP) [file pone.0310565.s003.zip › Raw files/NMR/Fig1C/moRNAtemp/10/pdata/1/thumb.png]

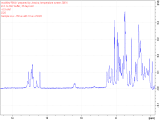

Supplement: S2 File — (ZIP) [file pone.0310565.s003.zip › Raw files/NMR/Fig1C/moRNAtemp/12/pdata/1/thumb.png]

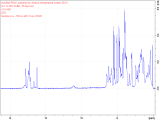

Supplement: S2 File — (ZIP) [file pone.0310565.s003.zip › Raw files/NMR/Fig1C/moRNAtemp/14/pdata/1/thumb.png]

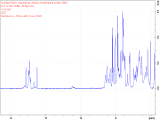

Supplement: S2 File — (ZIP) [file pone.0310565.s003.zip › Raw files/NMR/Fig1C/moRNAtemp/15/pdata/1/thumb.png]

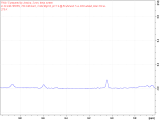

Supplement: S2 File — (ZIP) [file pone.0310565.s003.zip › Raw files/NMR/Fig1C/PNAtemp/10/pdata/1/thumb.png]

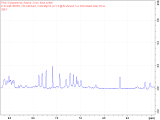

Supplement: S2 File — (ZIP) [file pone.0310565.s003.zip › Raw files/NMR/Fig1C/PNAtemp/12/pdata/1/thumb.png]

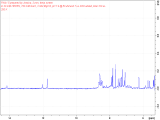

Supplement: S2 File — (ZIP) [file pone.0310565.s003.zip › Raw files/NMR/Fig1C/PNAtemp/14/pdata/1/thumb.png]
