# Supplementary material for: Peptide nucleic acids can form hairpins and bind RNA-binding proteins
Source: PLoS One. 2024 Sep 16;19(9):e0310565. doi: 10.1371/journal.pone.0310565 (PMC11404819; doi:10.1371/journal.pone.0310565)
Supplement: S1 File — (PDF) [file pone.0310565.s002.pdf]

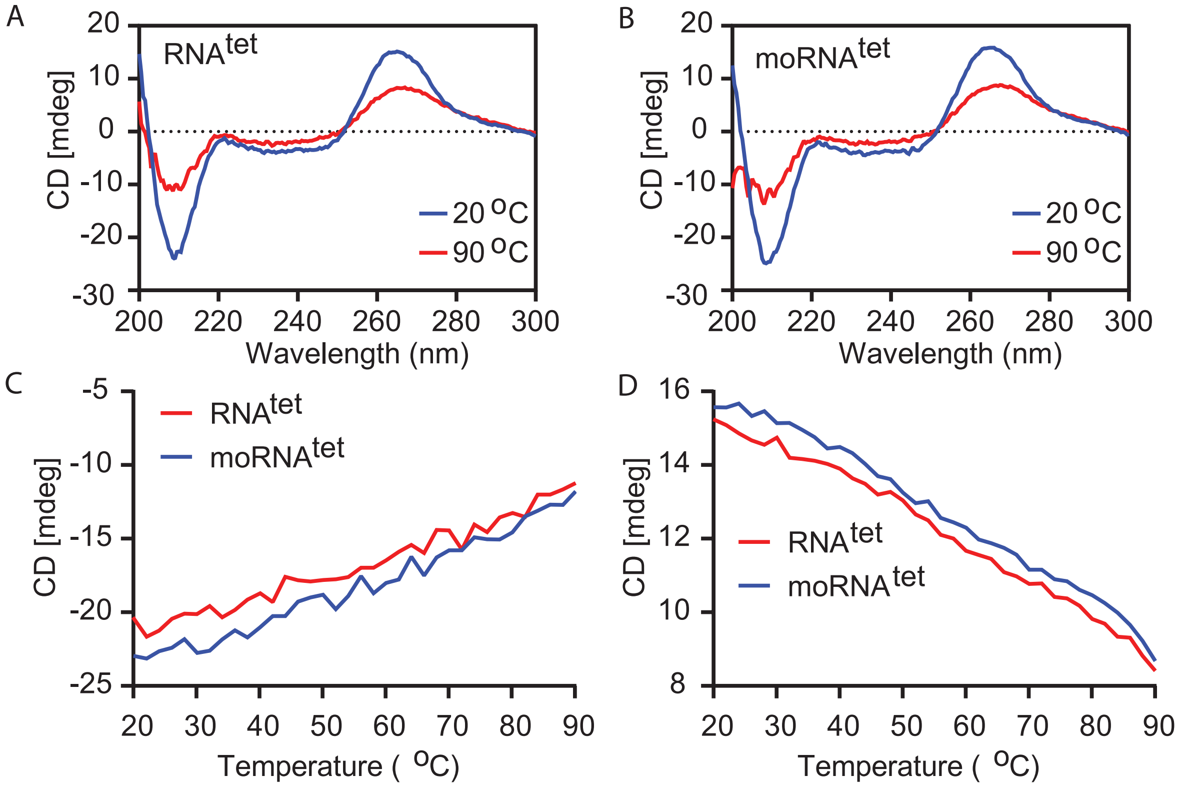

Supplementary Figure 1. CD spectra of RNA^{tet} (A) and moRNA^{tet} (B) at 20 and 90 ^{o}C. Thermal melt profiles of RNA^{tet} and moRNA^{tet} at 210 nm (C) and 265 nm (D).

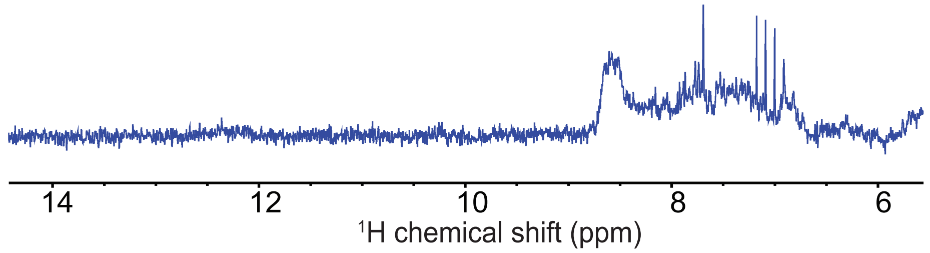

Supplementary Figure 2.1D ^{1}H NMR spectrum of PNA^{tet} at 25 ^{o}C before refolding showing no imino signals could be observed.

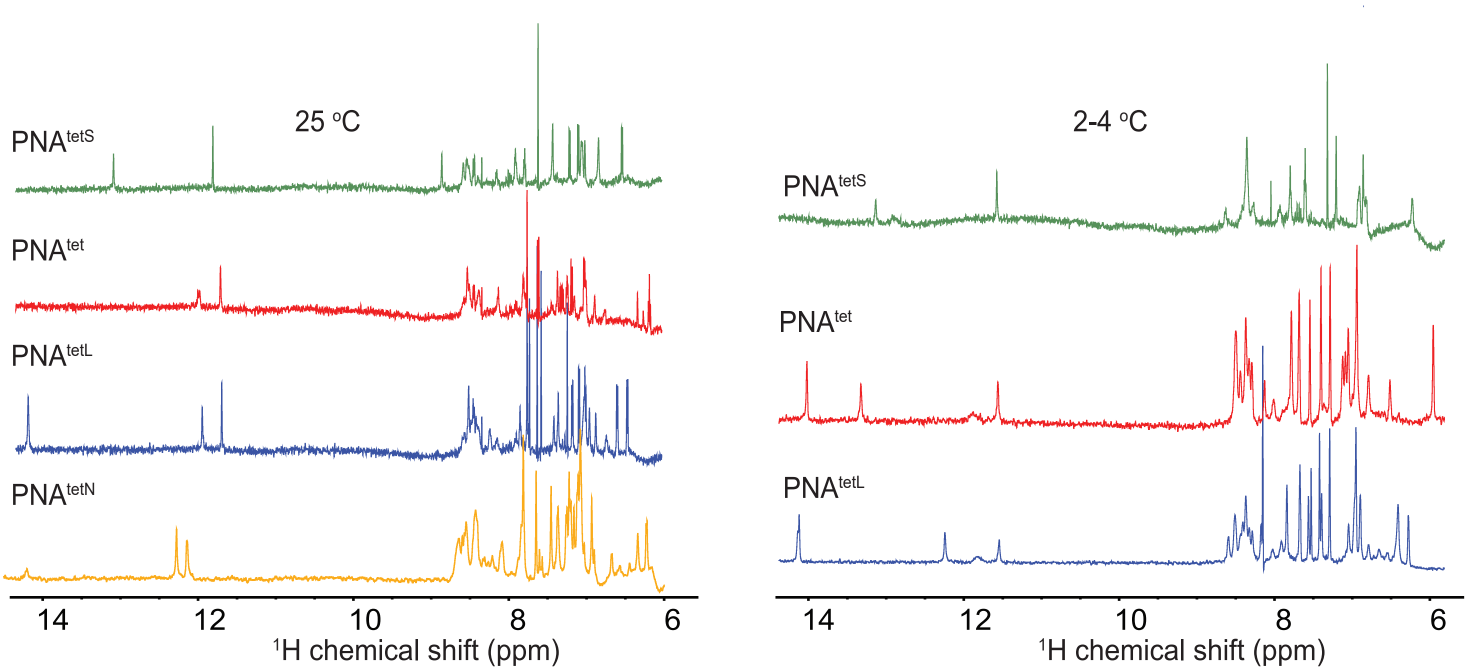

Supplementary Figure 3. 1D ^{1}H NMR spectra of PNA^{tetS}, PNA^{tet}, PNA^{tetL} and PNA^{tetN} at 25 ^{o}C and/or 2-4 ^{o}C. Clear imino peaks are seen in all PNAs at both temperatures with the number of imino signals increasing to match the maximum number of base pairs expected in the stems at the lower temperature.

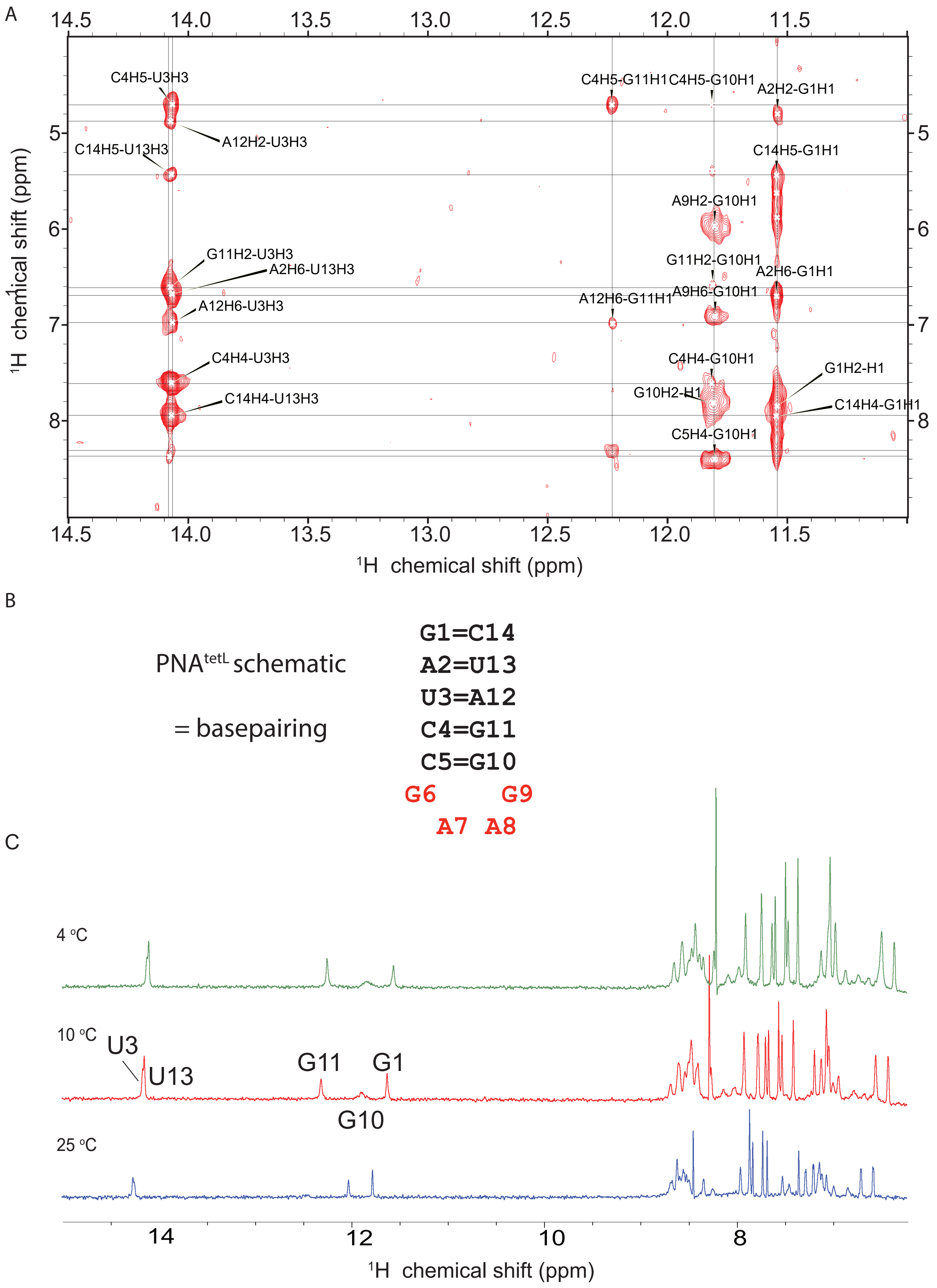

Supplementary Figure 4. A. Imino region of a 2D 1H-1H NOESY spectrum recorded on PNA^{tetL} at 10 °C with partial assignments. B. PNA^{tetL} schematic in proposed hairpin structure with numbering used in the assignments. C. 1D ^{1}H spectra of PNA^{tetL} at 4, 10 and 25 °C with imino protons labelled to their respective bases.

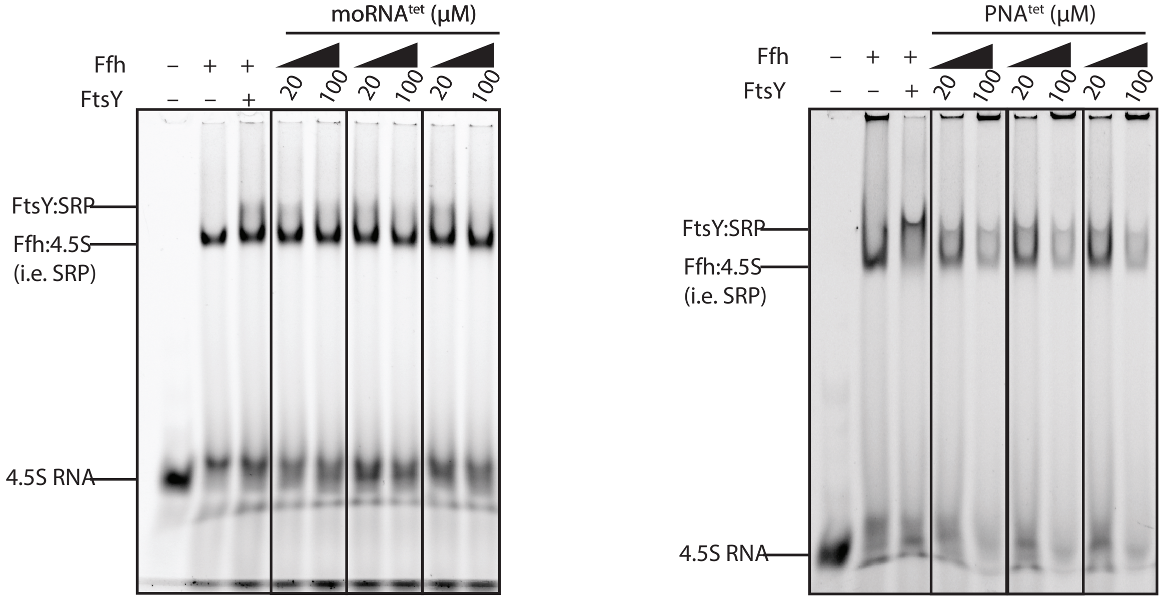

Supplementary Figure 5. EMSA gels showing moRNA^{tet} and PNA^{tet} inhibiting interaction between SRP and FtsY$_{NG}$. Cy5-labelled 4.5S RNA (20 nM) was pre-incubated with 200 nM Ffh and 1 mM GMP-PNP, before mixing with 400 nM FtsY$_{NG}$. Three sets of replicates of using FtsY$_{NG}$ pre-incubated with 20 μM or 100 μM PNA^{tet} or moRNA^{tet} were included. The samples were resolved on a 6% TBE gel after a 5-min incubation.

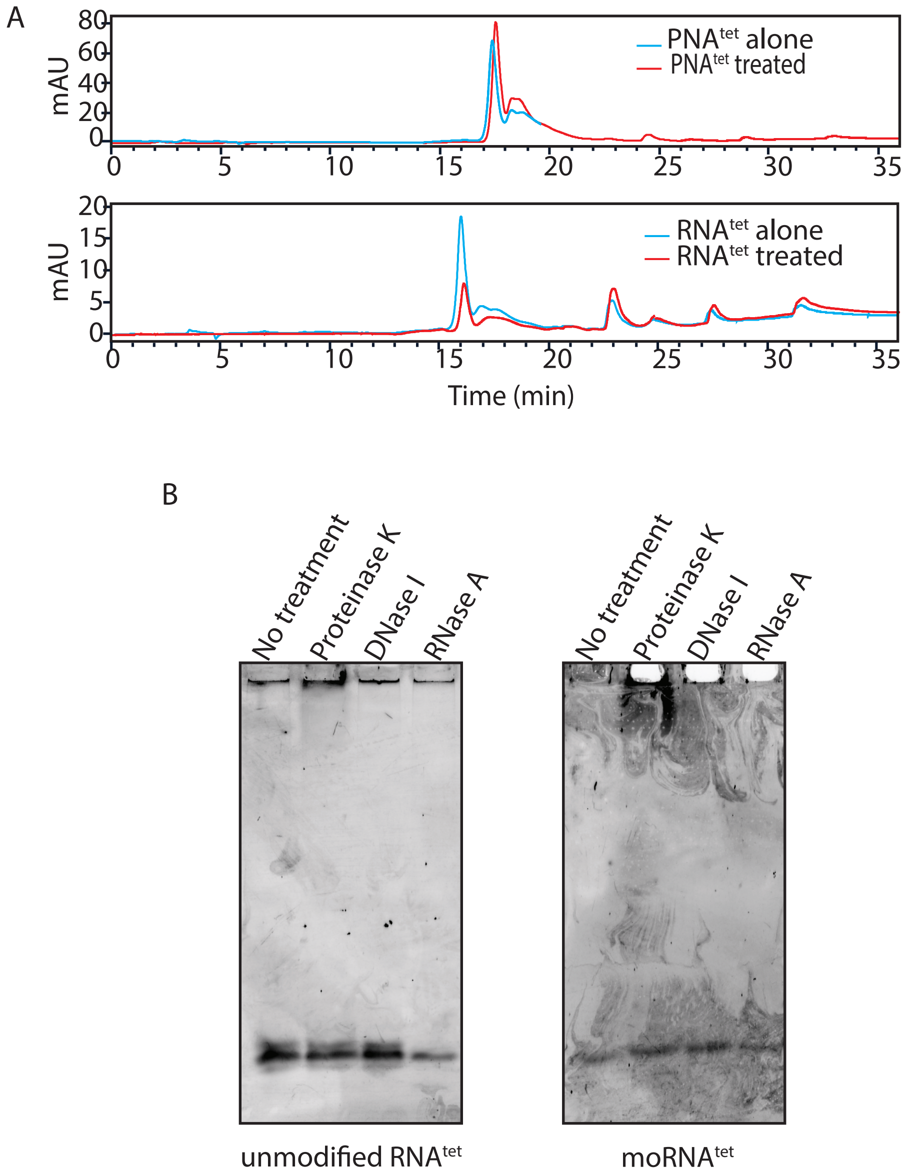

Supplementary Figure 6. (A) rpHPLC traces of 100 µM PNA^{tet} and 80 µM RNA^{tet} incubated at 37 °C with or without DNase, RNase and Proteinase K. The trace for PNA^{tet} alone terminated at ~20 min due to a power glitch. (B) Denaturing RNA gels showing moRNA^{tet} is more resistant to nuclease and protease attack than RNA^{tet}. Denaturing polyacrylamide gels used to assess nuclease stability of unmodified and modified RNA samples. RNA (5 µM) was digested with either nothing, Proteinase K (80 U/mL), DNase (0.3 mg/ml) or RNase A (0.3 mg/ml) for 1 h at 37°C. Samples were run on a 16% polyacrylamide gel containing 7 M urea using 1X TBE running buffer (90 mM Tris Base, 90 mM Boric acid, 2 mM EDTA, pH 8.3) for 25 min at 300 V. Gel stained with SYBR gold.

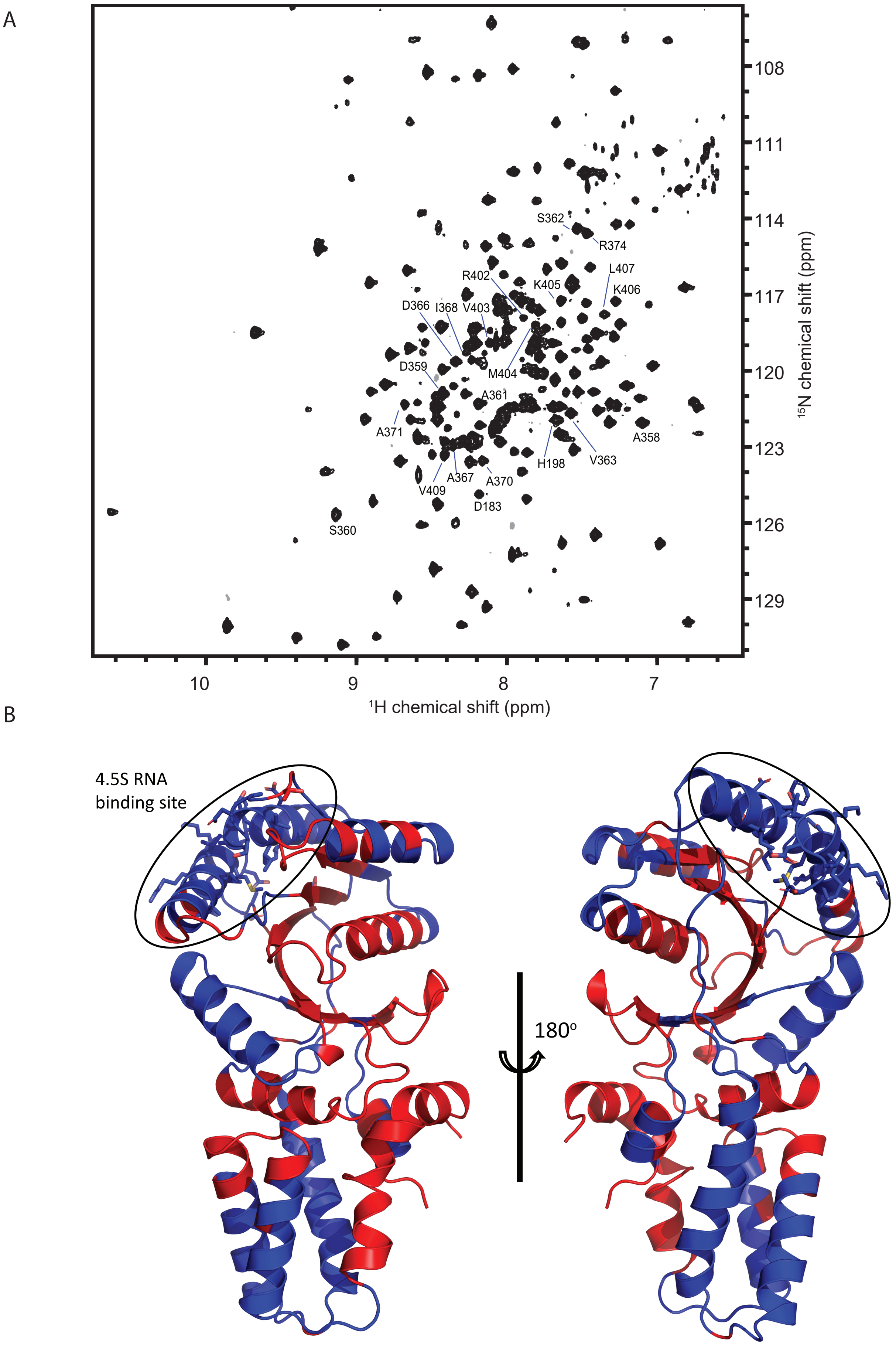

Supplementary Figure 7. Partial NMR assignments of $FtsY_{NG}$. A. TROSY-HSQC spectum of $^{2}H^{13}C^{15}N$-$FtsY_{NG}$ with assignments at the RNA-binding site indicated. B. Structure of $FtsY_{NG}$ (PDB: 6N6N). Assigned and unassigned residues are coloured in blue and red, respectively. RNA-binding site is circled with assigned RNA-binding site residues shown as sticks.

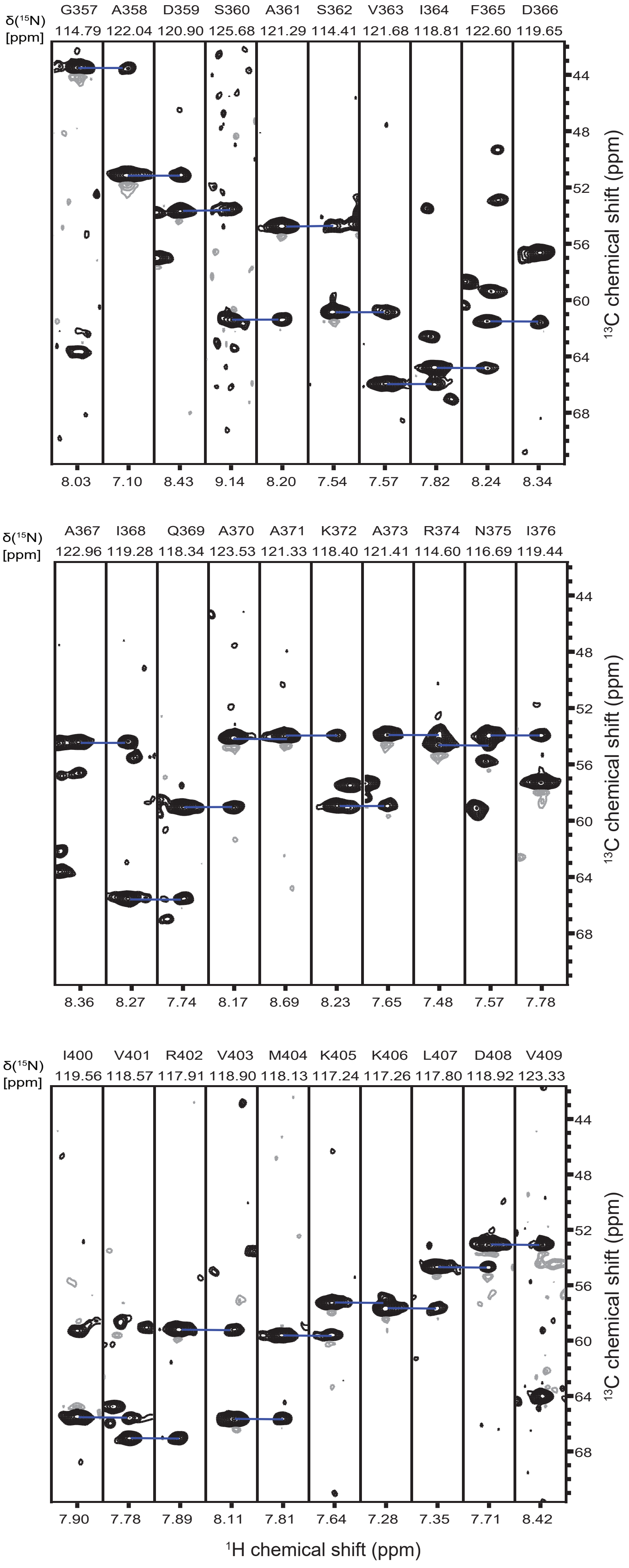

Supplementary Figure 8. Cα and Cα-1 connectivity for several residues at the RNA binding site is shown. HN-Cα strips (A-C) extracted from the 3D TROSY-HNCA spectrum. δ(^{15}N) indicates the ^{15}N chemical shift of the stripe.

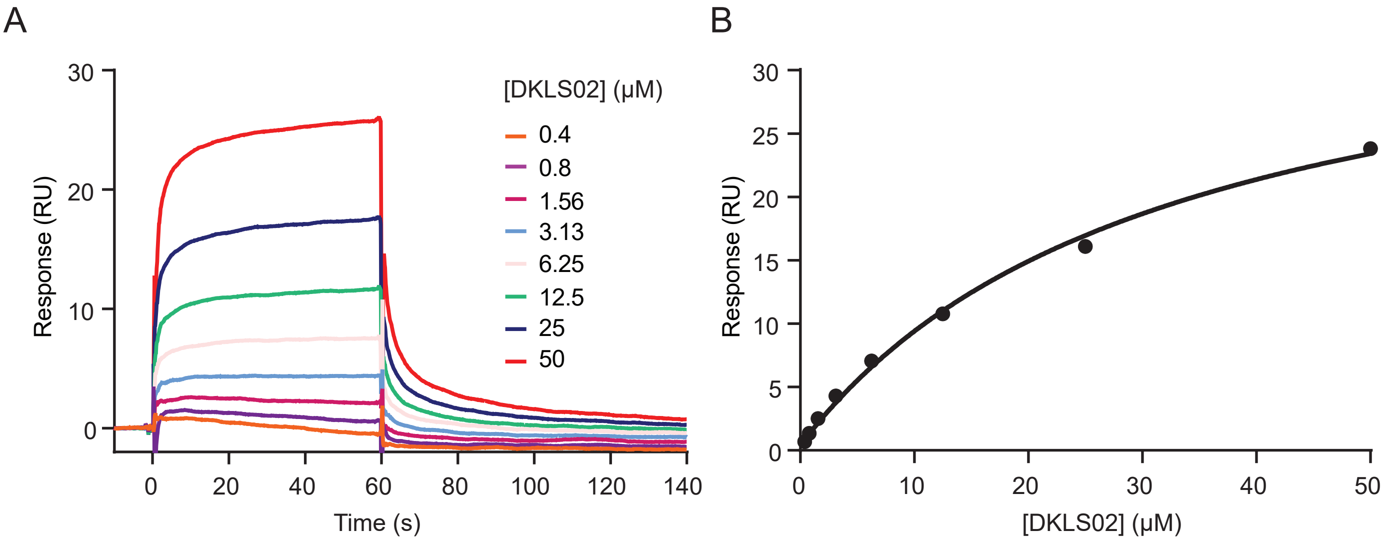

Supplementary Figure 9. Nsp9 binds to DKLS02 modified RNA hairpin with a K_D of ~30 μM. SPR sensorgram and fit to equilibrium response shown for binding to immobilised Avi-Nsp9.
