# Supplementary material for: Peptide nucleic acids can form hairpins and bind RNA-binding proteins
Source: PLoS One. 2024 Sep 16;19(9):e0310565. doi: 10.1371/journal.pone.0310565 (PMC11404819; doi:10.1371/journal.pone.0310565)
Supplement: S2 File — (ZIP) [file pone.0310565.s003.zip › Raw files/CD/moRNA CD methods.docx]

Circular Dichroism (CD)

All CD spectra were measured on a JASCO J-815 CD Spectrometer using quartz cuvettes with 0.1 cm path length. Spectra from 200 to 300 nm were recorded at both 20 ^o^C and 90 ^o^C, averaged over three scans and collected at a rate of 50 nm/min (1.00 nm bandwidth, 1 sec D.I.T). A melt curve from 20 ^o^C to 90 ^o^C was recorded at 265 nm and 210 nm with a ramp of 1 ^o^C/min, collected in 2 ^o^C intervals (1.00 nm bandwidth, 4 sec D.I.T). CD samples were prepared by refolding 30 μM RNA^tet^ and moRNA^tet^ in 10 mM HEPES, pH 7.0 and 0.5 mM MgCl_2_.
