# Supplementary material for: Peptide nucleic acids can form hairpins and bind RNA-binding proteins
Source: PLoS One. 2024 Sep 16;19(9):e0310565. doi: 10.1371/journal.pone.0310565 (PMC11404819; doi:10.1371/journal.pone.0310565)
Supplement: S2 File — (ZIP) [file pone.0310565.s003.zip › Raw files/HPLC/rpHPLC_PNA_alone.pdf]

HPLC report

221221_PNA_alone

Data file: C:\Users\Public\Documents\ChemStation\2\Data\Rezwan\2022-12-22 (09-26-14) 221221_PNA_alone_Run_1.D

Sample name: 221221_PNA_alone

Description: PNA in 1 mL Buffer A

**Instrument:**		**Injection:**	1 of 1
**Injection date:**	2022-12-22 09:26:29+11:00	**Last changed:**	2022-12-30 18:08:36+11:00
**Acq. method:**	PNA_B5to90_30min_timefrac_ann.M		
**Analysis method:**	PNA_B5to90_30min.M		

Signals overlaid:

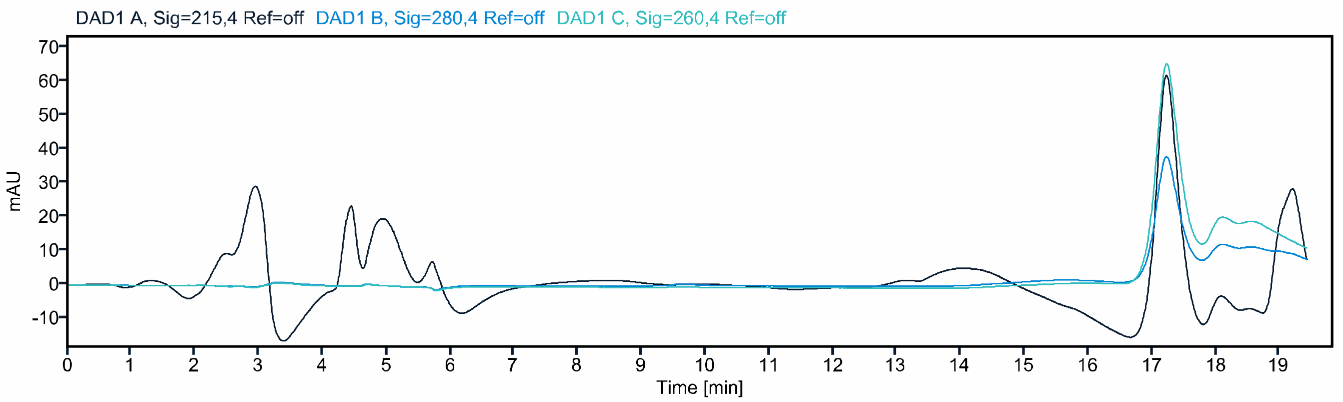

Signals separated:

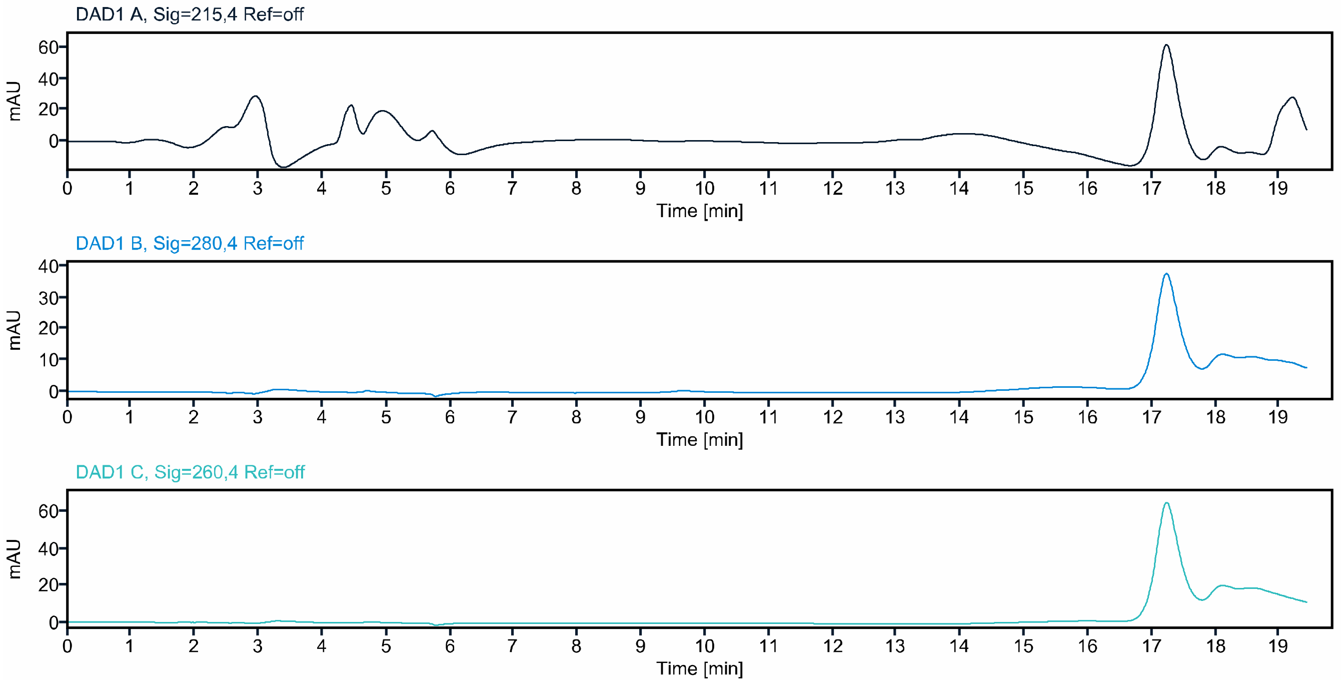

No Data Found!
