# Supplementary material for: Peptide nucleic acids can form hairpins and bind RNA-binding proteins
Source: PLoS One. 2024 Sep 16;19(9):e0310565. doi: 10.1371/journal.pone.0310565 (PMC11404819; doi:10.1371/journal.pone.0310565)
Supplement: S2 File — (ZIP) [file pone.0310565.s003.zip › Raw files/HPLC/rpHPLC_PNA_RDP.pdf]

HPLC report

221221_PNA_RDP

Data file: C:\Users\Public\Documents\ChemStation\2\Data\Rezwan\2022-12-22 (12-52-19) 221221_PNA_RDP_Run_1.D

Sample name: 221221_PNA_RDP

Description: PNA digested with RNase, DNase and Protease K in 1 mL Buffer A

**Instrument:**	HPLC	**Injection:**	1 of 1
**Injection date:**	2022-12-22 12:52:47+11:00	**Last changed:**	2022-12-30 18:08:36+11:00
**Acq. method:**	PNA_B5to90_30min.M		
**Analysis method:**	PNA_B5to90_30min.M		

Signals overlaid:

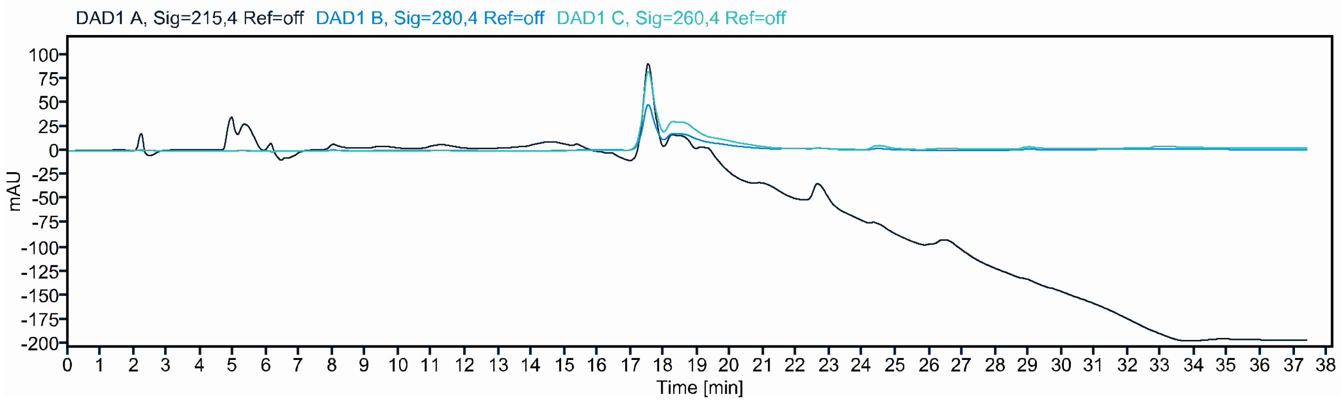

Signals separated:

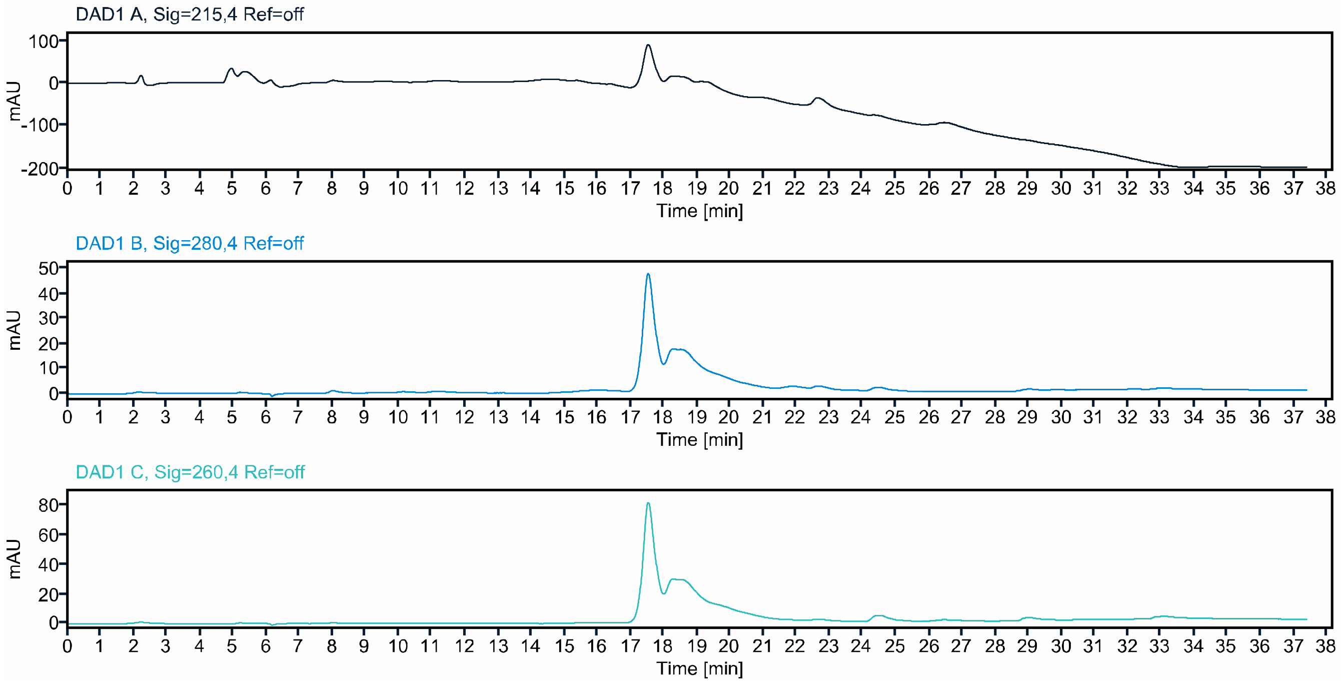

Acquisition Method: PNA_B5to90_30min.M
Path: C:\Users\Public\Documents\ChemStation\2\Methods\Rezwan

Acquisition Method

Fraction Collector (G1364C)

Peak Detector Mode:	at least one peak detector
Rinse Needle At Start Of Collection:	No
Rinse Needle Between Collection:	No

Fill Volume

Fill Volume Mode: As Configured

Fraction Trigger Mode

Fraction Trigger Mode: Off

Peak Detectors

Peak Detector

Detector type with serial number	Down Slope	Mode	Threshold	Unit	Upper Threshold	Up Slope
G1315D:DEAAX06293	5.00	Off	5.000	mAU	3000.000	5.00

Stoptime

Stoptime Mode: As Pump/Injector

Posttime

Posttime Mode: Off

DAD (G1315D)

Peakwidth:	> 0.1 min (2 s response time) (2.5 Hz)
Slit:	4 nm
UV Lamp Required:	Yes
Vis Lamp Required:	Yes

Analog Output 1

Analog 1 Zero Offset:	5 %
Analog 1 Attenuation:	1000 mAU

Analog Output 2

Analog 2 Zero Offset:	5 %
Analog 2 Attenuation:	1000 mAU

Signals

Signal table

Acquire	Signal	Wavelength (nm)	Bandwidth (nm)	Use Ref.
Yes	Signal A	215	4	No
Yes	Signal B	280	4	No
Yes	Signal C	260	4	No
No	Signal D			
No	Signal E			
No	Signal F			
No	Signal G			
No	Signal H			

Prepare Mode

Margin for negative Absorbance: 100 mAU

Autobalance

Autobalance Prerun: Yes
Autobalance Postrun: No

Spectrum

Spectrum Store: None

Stoptime

Stoptime Mode: As Pump/Injector

Posttime

Posttime Mode: Off

Binary Pump (G1312A)

Flow: 1.000 mL/min
Low Pressure Limit: 0.000 MPa
High Pressure Limit: 15.000 MPa
Maximum Flow Gradient: 100.000 mL/min²

Solvent Composition

Channel	Name 1	Used	Percent (%)
A		Yes	95.0
B		Yes	5.0

Timetable

Time (min)	A (%)	B (%)	Flow (mL/min)	Pressure (MPa)
Start. Cond.	95.0	5.0	1.000	15.000
2.00	95.0	5.0	1.000	15.000
22.00	10.0	90.0	1.000	15.000
27.00	10.0	90.0	1.000	15.000
30.00	95.0	5.0	1.000	15.000

Stroke A

Automatic Stroke Calculation A: Yes

Stroke B

Automatic Stroke Calculation B: Yes

Compress A

Compressibility Mode A: Compressibility Value Set
Compressibility A: 50 10e-6/bar

Compress B

Compressibility Mode B: Compressibility Value Set
Compressibility B: 115 10e-6/bar

Stoptime

Stoptime Mode: Time set
Stoptime: 35.00 min

Posttime

Posttime Mode: Off
