# Supplementary material for: Peptide nucleic acids can form hairpins and bind RNA-binding proteins
Source: PLoS One. 2024 Sep 16;19(9):e0310565. doi: 10.1371/journal.pone.0310565 (PMC11404819; doi:10.1371/journal.pone.0310565)
Supplement: S3 File — (DOCX) [file pone.0310565.s004.docx]

Figure 1C. NMR data.

Refer to …\Raw files\NMR\Fig1C\PNAtemp

/10 2 ^o^C

/12 12 ^o^C

/14 20 ^o^C

/15 25 ^o^C

Refer to …\Raw files\NMR\Fig1C\moRNAtemp

/10 2 ^o^C

/12 12 ^o^C

/14 20 ^o^C

/15 25 ^o^C

Figure 1D. DLS of 50 µM refolded PNA^tet^

| Diameter(nm) | % Number |
| --- | --- |
| 0.0401934 | 0 |
| 0.0532214 | 0 |
| 0.0704723 | 0 |
| 0.0933148 | 0 |
| 0.123561 | 0 |
| 0.163612 | 0 |
| 0.216644 | 0 |
| 0.286865 | 0 |
| 0.379848 | 0 |
| 0.50297 | 0 |
| 0.665999 | 0 |
| 0.881872 | 0 |
| 1.16772 | 0 |
| 1.54621 | 0 |
| 2.04739 | 0 |
| 2.71102 | 15.8745 |
| 3.58976 | 84.1255 |
| 4.75332 | 0 |
| 6.29403 | 0 |
| 8.33414 | 0 |
| 11.0355 | 0 |
| 14.6125 | 0 |
| 19.3489 | 0 |
| 25.6205 | 0 |
| 33.925 | 0 |
| 44.9213 | 0 |
| 59.4818 | 0 |
| 78.7618 | 0 |
| 104.291 | 0 |
| 138.095 | 0 |
| 182.857 | 0 |
| 242.127 | 0 |
| 320.609 | 0 |
| 424.529 | 1.30E-10 |
| 562.133 | 1.02E-10 |
| 744.339 | 0 |

Figure 2A raw data.

For raw data and binding curve fitting values, refer to “MST raw data and fitting curves. xlsx”.

Figure 2B raw data.

Individual KD of each replicate and folds of affinity changes in presence of PNA^tet^, PNA^tetS^, PNA^tetL^ and PNA^tetN^, relative to the average affinity of “no PNA” control.

| **vs. PNA1.5 and 4** | |  |  | **vs. PNA1.5 and 4** | |  |  |
| --- | --- | --- | --- | --- | --- | --- | --- |
| replicates | no PNA | 5 uM PNAtetL |  | replicates | no PNA | 5 uM PNAtetL |  |
| 1 | 112.54 | 371.34 |  | 1 | 1.01 | 3.33 |  |
| 2 | 132.285 | 295.24 |  | 2 | 1.19 | 2.65 |  |
| 3 | 89.502 | 317.29 |  | 3 | 0.80 | 2.85 |  |
| average | **111.44** |  |  |  |  |  |  |
|  |  |  |  |  |  |  |  |
| **vs. PNA3 and 9** | |  |  | **vs. PNA3 and 9** | |  |  |
| replicates | no PNA | 5 uM PNAtetS | 5 uM PNAtetN | replicates | no PNA | 5 uM PNAtetS | 5 uM PNAtetN |
| 1 | 315.03 | 256.76 | 241.51 | 1 | 0.87 | 0.71 | 0.67 |
| 2 | 403.54 | 170.5 | 352.49 | 2 | 1.11 | 0.47 | 0.97 |
| 3 | 368.42 | 258.2 | 437.62 | 3 | 1.02 | 0.71 | 1.21 |
| average | **362.33** |  |  |  |  |  |  |
|  |  |  |  |  |  |  |  |
| **vs. PNA1.5** | |  |  | **vs. PNA1.5** | |  |  |
| replicates | no PNA | 5 uM PNAtet |  | replicates | no PNA | 5 uM PNAtet |  |
| 1 | 78.002 | 207.97 |  | 1 | 0.94 | 2.51 |  |
| 2 | 87.292 | 207.82 |  | 2 | 1.05 | 2.50 |  |
| 3 | 83.681 | 247.13 |  | 3 | 1.01 | 2.98 |  |
| average | **82.99** |  |  |  |  |  |  |


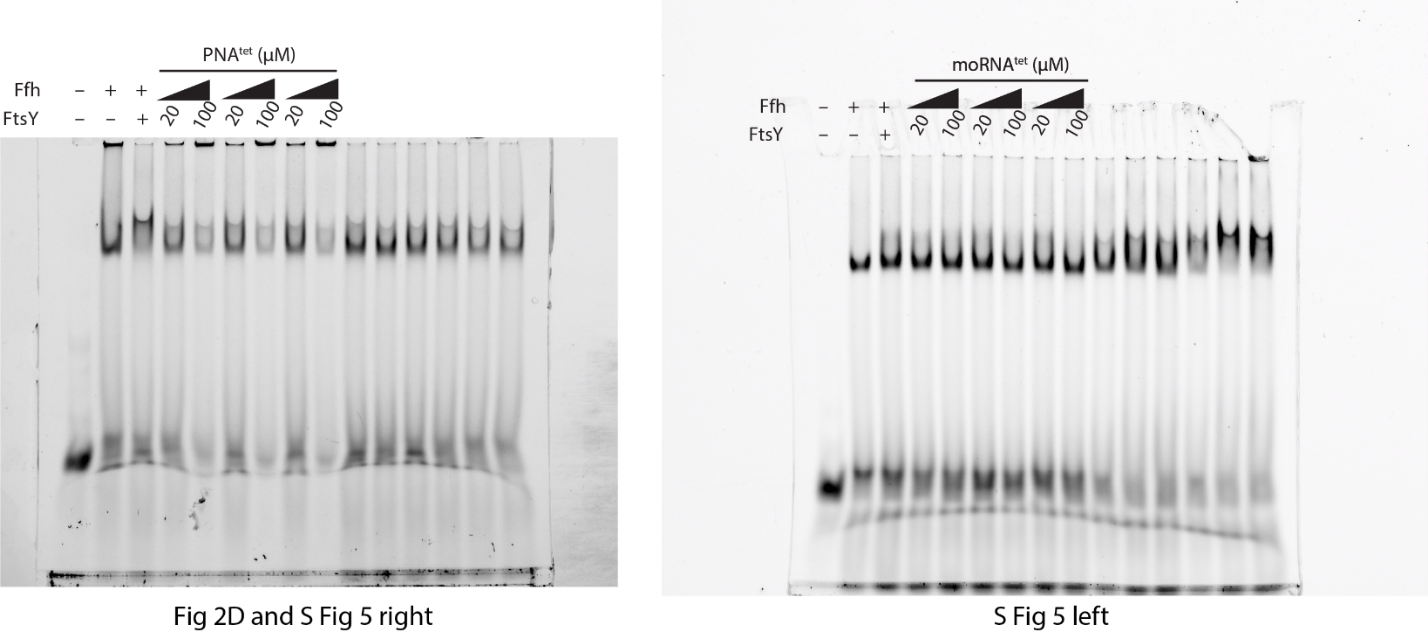
Figure 2C, SFig5 uncropped EMSA gel image.

Figure 3A-E. SPR data

refer to …\Raw files\MST and SPR data\Raw_Data_For_All_SPR_Figures

Figure 3F-G. NMR data

refer to …\Raw files\NMR\Fig3FG

Figure 4A-B. SPR data

refer to …\Raw files\MST and SPR data\Raw_Data_For_All_SPR_Figures

Figure 4C. NMR data

refer to …\Raw files\NMR\Fig4C\ak220119_Nsp9_PNA

/12 Nsp9 alone

/14 Nsp9 + PNA^tet^ 1:1

SFig 1A. CD spectra

…\Raw files\CD

SFig 2-4. NMR data

SFig 2, refer to …\Raw files\NMR\Sfig2

SFig 3, refer to …\Raw files\NMR\Sfig3

SFig 4, refer to …\Raw files\NMR\SFig4_NOESY PNA

SFig 5. uncropped EMSA gel images are shown above.

SFig 5A. HPLC profile

refer to …\Raw files\HPLC


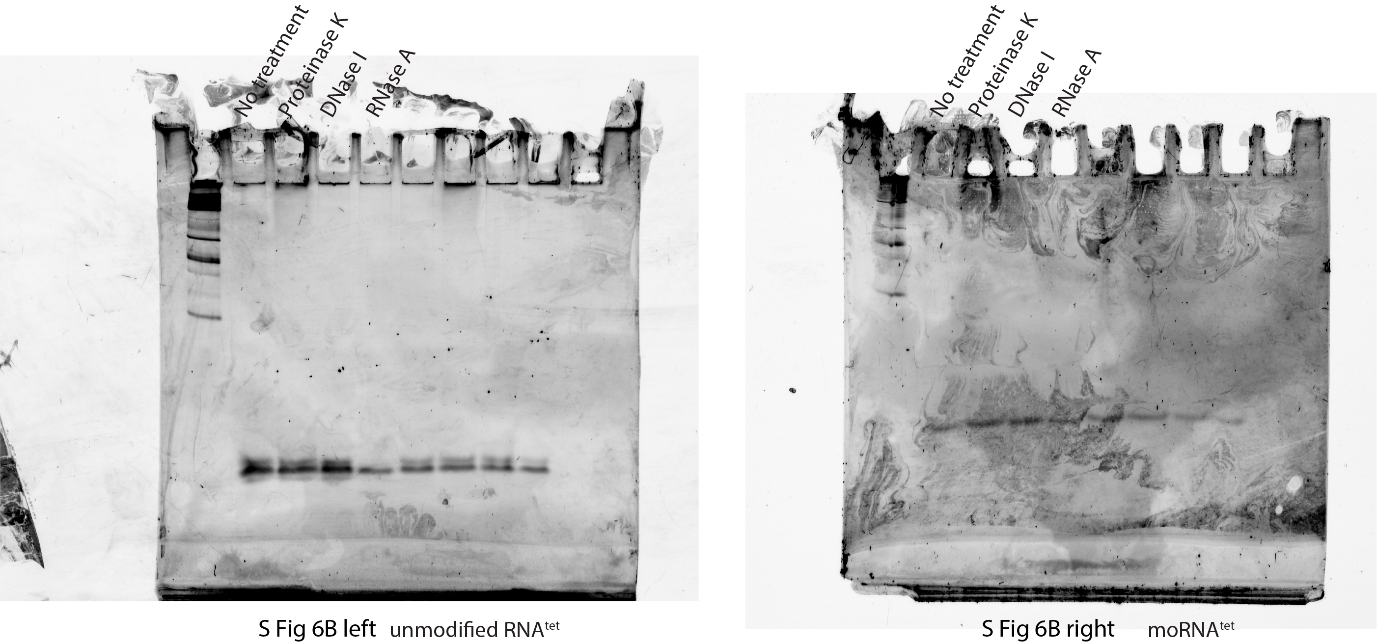
SFig 5B. uncropped gel image:

SFig 7A & 8. NMR data

BMRB deposition 52588

refer to …\Raw files\NMR\Sfig78

SFig 9. SPR data.

refer to …\Raw files\MST and SPR data\Raw_Data_For_All_SPR_Figures
