# Supplementary material for: Peptide nucleic acids can form hairpins and bind RNA-binding proteins
Source: PLoS One. 2024 Sep 16;19(9):e0310565. doi: 10.1371/journal.pone.0310565 (PMC11404819; doi:10.1371/journal.pone.0310565)
Supplement: S2 File — (ZIP) [file pone.0310565.s003.zip › Raw files/NMR/Sfig3/PNA_all3_277K.pdf]

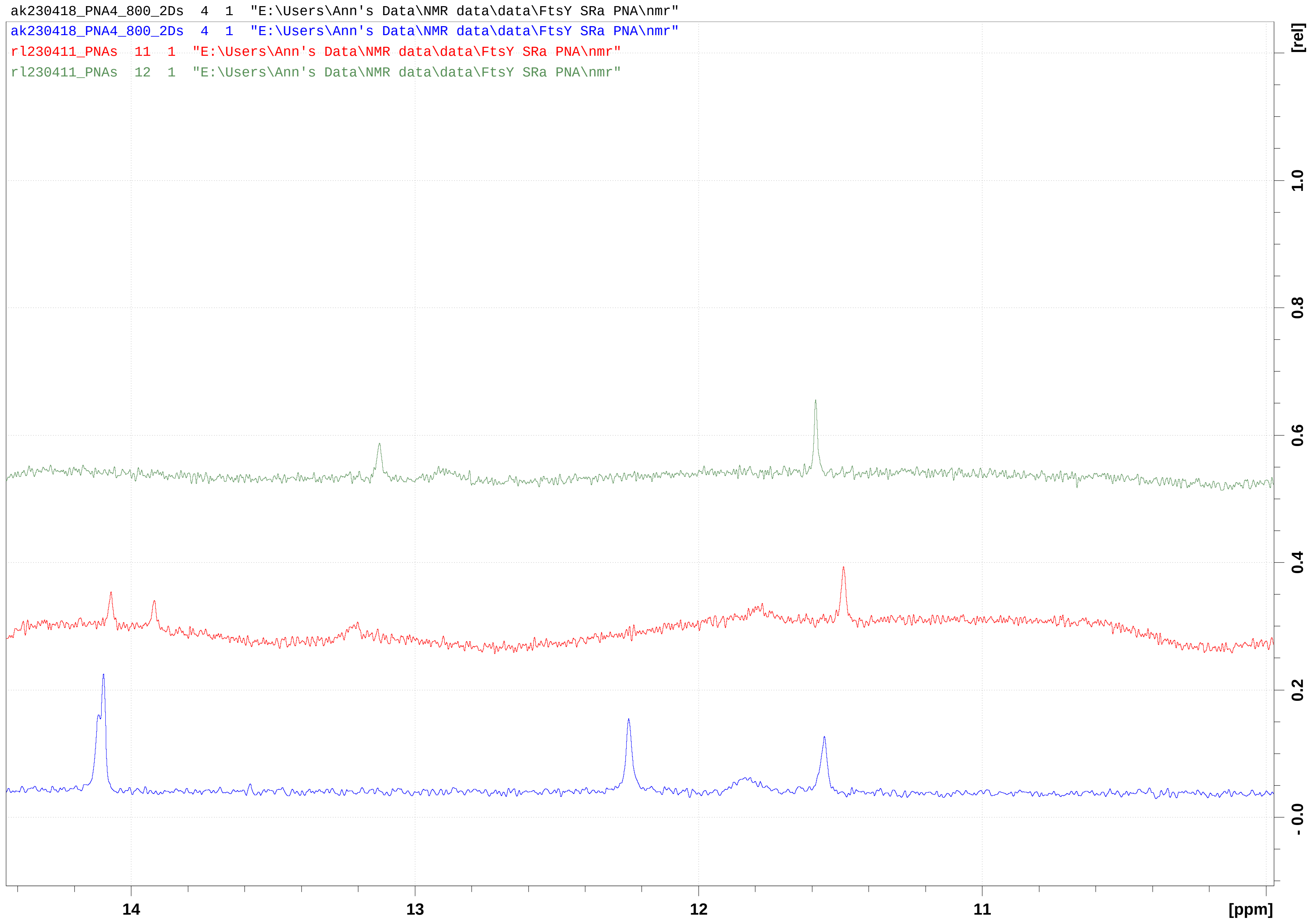
ak230418_PNA4_800_2Ds 4 1 "E:\Users\Ann's Data\NMR data\data\FtsY SRa PNA\nmr"
ak230418_PNA4_800_2Ds 4 1 "E:\Users\Ann's Data\NMR data\data\FtsY SRa PNA\nmr"
rl230411_PNAs 11 1 "E:\Users\Ann's Data\NMR data\data\FtsY SRa PNA\nmr"
rl230411_PNAs 12 1 "E:\Users\Ann's Data\NMR data\data\FtsY SRa PNA\nmr"
[rel]
1.0
0.8
0.6
0.4
0.2
- 0.0
14
13
12
11
[ppm]
