# Supplementary material for: Peptide nucleic acids can form hairpins and bind RNA-binding proteins
Source: PLoS One. 2024 Sep 16;19(9):e0310565. doi: 10.1371/journal.pone.0310565 (PMC11404819; doi:10.1371/journal.pone.0310565)
Supplement: S2 File — (ZIP) [file pone.0310565.s003.zip › Raw files/NMR/Sfig3/PNA_all3_298_6-14ppm.pdf]

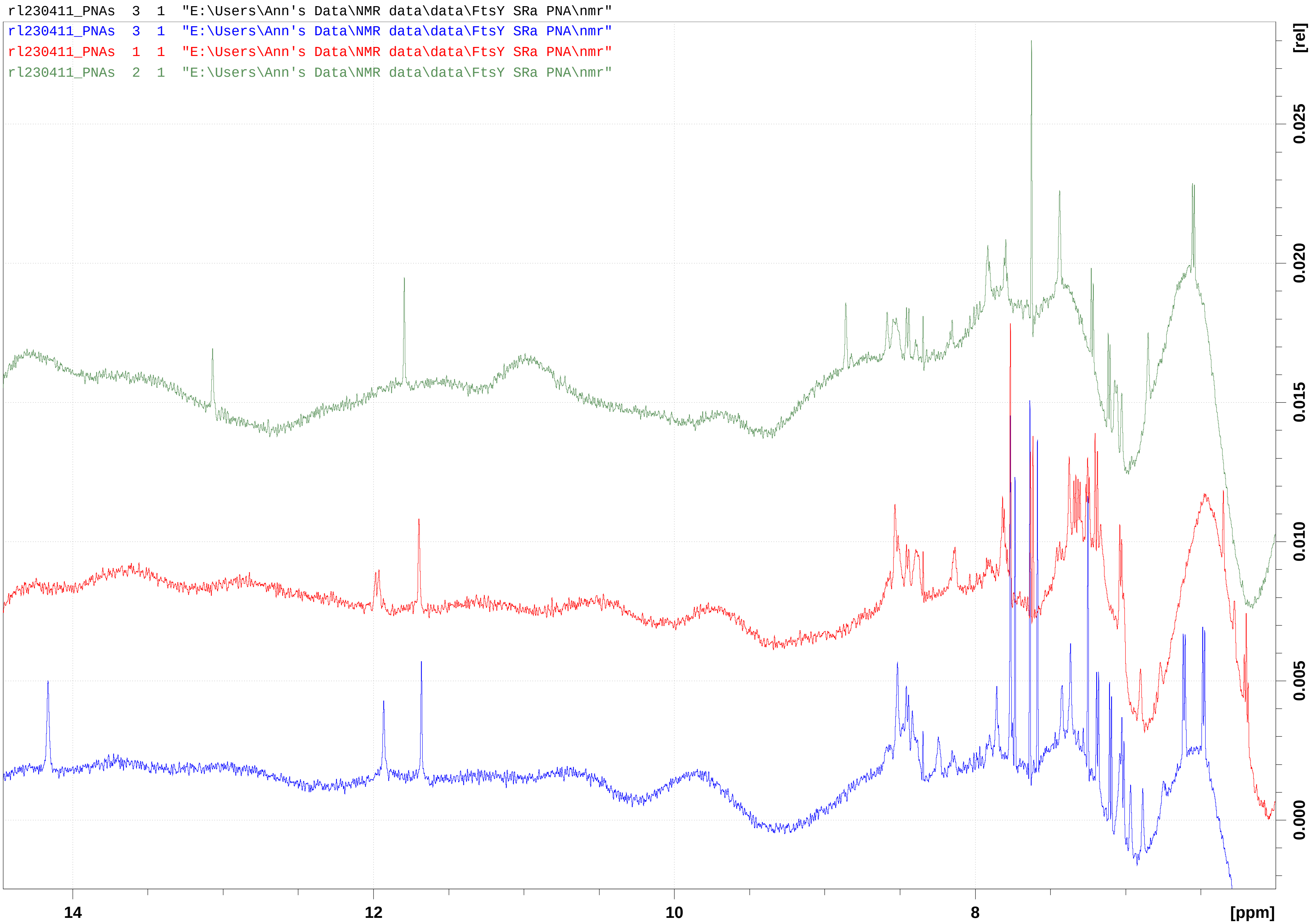
rl230411_PNAs 3 1 "E:\Users\Ann's Data\NMR data\data\FtsY SRa PNA\nmr"
rl230411_PNAs 3 1 "E:\Users\Ann's Data\NMR data\data\FtsY SRa PNA\nmr"
rl230411_PNAs 1 1 "E:\Users\Ann's Data\NMR data\data\FtsY SRa PNA\nmr"
rl230411_PNAs 2 1 "E:\Users\Ann's Data\NMR data\data\FtsY SRa PNA\nmr"
[rel]
0.025
0.020
0.015
0.010
0.005
0.000
14
12
10
8
[ppm]
