# Supplementary material for: Peptide nucleic acids can form hairpins and bind RNA-binding proteins
Source: PLoS One. 2024 Sep 16;19(9):e0310565. doi: 10.1371/journal.pone.0310565 (PMC11404819; doi:10.1371/journal.pone.0310565)
Supplement: S2 File — (ZIP) [file pone.0310565.s003.zip › Raw files/NMR/SFig78/FtsY_assignments.docx]

**FtsY assignments**

**Unambiguous assignments:**

The H, N, CA, and CB assignments for the following segments of the FtsY were double-checked, and they are unambiguous.

R25-L37, K53-L67, D69-E83, L92-A98, F123-S129, A142-Q145, R152-I155, S166-S168, D172-D183, H198-L203, R208-E216, T229-S235, K238-G245, T247-G248, G271-I273, R275-Y276, K290-D293, E296-L298

**Residues having ambiguous assignments:**

E39, Q40, L41, I84, A86, K87, V88, D89, E90, W149, G150, Q151, V157, I158, A159, Q160, G163, A164, D165, V169, I170, F171, T189, A190, G191, R192, S197, K205, I206, V207, A217, Q236, A237, L246, F270, I274, I277

**Reference offsets are required to analyse the following 2D and 3D NMR spectra:**

TROSY_HSQC (Bruker format)

**HN -> + 0.0 ppm; N -> +0.0 ppm**

HNCA (UCSF format)

**HN -> + 0.0 ppm; N -> +0.0 ppm; CA -> + 2.7 ppm**

HNcoCA (UCSF format)

**HN -> + 0.0 ppm; N -> +0.0 ppm; CA -> + 2.7 ppm**

HNcoCACB (UCSF format)

**HN -> + 0.0 ppm; N -> +0.0 ppm; Carbon -> + 2.7 ppm**

HNCACB_Aug22 (UCSF format)

**HN -> - 0.083 ppm; N -> -0.059 ppm; Carbon -> 0.0 ppm**

HNCB_Aug22 (UCSF format)

**HN -> - 0.072 ppm; N -> -0.059 ppm; Carbon -> 0.0 ppm**
