# Supplementary material for: Peptide nucleic acids can form hairpins and bind RNA-binding proteins
Source: PLoS One. 2024 Sep 16;19(9):e0310565. doi: 10.1371/journal.pone.0310565 (PMC11404819; doi:10.1371/journal.pone.0310565)
Supplement: Attachment — Submitted filename: Response to Reviewers.docx [file pone.0310565.s005.docx]

|  | |  |  |
| --- | --- | --- | --- |
|  | ABN 15 211 513 464 | |  |
|  | **A/Prof Ann Kwan**  School of Life and Environmental Science and Sydney Nano  Email: [ann.kwan@sydney.edu.au](mailto:ann.kwan@sydney.edu.au)  **Dr Sandro Ataide**  School of Life and Environmental Science  Email: [sandro.ataide@sydney.edu.au](mailto:sandro.ataide@sydney.edu.au) | |  |

15th August 2024

Dear Mauricio,

Thanks for considering and reviewing our manuscript “Peptide Nucleic Acids can form hairpins and bind RNA-binding proteins”.

We thank the reviewers for their thoughtful comments and suggestions and our point-by-point responses are below.

We hope you will find our revised manuscript suitable for publication in PLOS ONE. As requested, we have submitted a marked up copy and a cleaned copy.

Yours sincerely,

A/Prof Ann Kwan and Dr Sandro Ataide.

**Editorial comments summarising comments from Reviewer 1 and 2:**

Reviewer 1’s comments:

*1. Add a discussion of the future directions of how to improve the PNA/protein binding strength and specificity. In particular, what kind of PNA SL modifications: structural, chemical, sequence (?) could make it a better competitor for the cognate RNA SL?*

We agree with the reviewer that the strength of PNA/protein interaction is strongly affected by the length of the PNA stem, the sequence of its loop, type of the PNA or modifications used. The longer PNA performed better in competition assays, but at the cost of solubility. We have expanded on the discussion about general strategies to improve PNA solubility in the manuscript text. Please refer to the section from Lines 547 to 565.

The other strategy to improve binding affinity is by optimising the binding sequence. In Lines 573 to 586, we describe how SELEX and affinity distribution analysis may be used to find better binders. Another way to improve the PNA-Protein binding affinity is via rational design strategies using high resolution structures of the PNA-protein complex. We have attempted to crystalise the complex but have not had success so far and we suspect this is at least partially due to the limited PNA solubility and the requirement for a high concentration of PNA-protein to form crystals. It is very possible that further chemical modifications to the PNA backbone to allow H-bonding with the protein and expanding those interactions with additional residues can help. Unfortunately for this study, we are limited to a small number of commercially available modifications even though PNA modifications to enhance solubility and functions are a hot area of research with several recent publications. We are hopeful that our findings would expand the interest and demand for PNAs and this would further drive research in PNA modification and synthesis. We have recently set up a collaboration with Dr Emma Watson, University of Adelaide, who has embarked on improving PNA capabilities in her new laboratory following on from her postdoctoral work:

<https://onlinelibrary.wiley.com/doi/full/10.1002/hlca.202300110>

Reviewer 2’s comments:

*2. It is not clear what role glutamate has in binding, if any at all. Please explain it.*

The glutamate was added to the PNA at the tetraloop to mimic the phosphate from the 4.5SRNA backbone that interacts with a positive patch of amino acids from the FtsY. To test the effect of this glutamate, we have used a PNA^tet^ variant without glutamate in MST and SPR assays. However, this variant aggregated on the SPR surface and produced noisy and uninterpretable MST results. We also tested a glutamate-free variant of PNA^tetN^ and observed similar results. It is likely this glutamate also serves as a solubility and/or stability modification to the PNA. Note that PNA^tetN^, which retains the glutamate but with a different tetraloop sequence did not inhibit the FtsY and RNA interaction, indicating that the loop sequence is the main contributor.

*3. What is the OO linker?*

The OO linker is a 2-aminoethoxy-2-ethoxy acetic acid (AEEA) linker. OO linker is the name used by the manufacturer, and we have changed it to AEEA linker in the revised manuscript. It was added to increase the solubility of PNA.

*4. Fig 2A: why are the three black lines different in the three panels?*

The variations in K_D_ observed are likely due to different batches of purified protein and/or RNA. In particular, the fluorescently labelled RNA degrades easily, and this is a major motivation for using PNAs as RNA mimics. The K_D_ between FtsY and RNA is typically ~100−200 μM , but occasionally it has been observed to increase to ~300 μM like in panel 3. The experiments reported in panel 3 were performed a few months after those shown in the first two panels as the different PNAs were ordered and available at different times. Therefore, we have chosen to compare relative rather than absolute binding affinities in the MST assays. In this study, the affinities measured in the presence and absence of PNAs were performed on the same day using the same aliquot of protein and RNA and the ratio of affinity was presented.

Additional comment from Review 2 following on the above question

*As Ffh is also well-known to bind to RNA, the authors must check whether Ffh-RNA binding can be similarly inhibited by the synthesized PNA.*

PNA^tet^ and its variants would not be able to inhibit the Ffh-4.5S RNA interaction because that interaction is much stronger and is mediated via the M domain and not the S-domain tetraloop. The M domain that binds Ffh is an asymmetrical loop located in another part of the stem and is surrounded by a different structure and sequence.

*5. Line 472: It is not clear what the authors mean by “using GDP binding as a positive control”. Do they mean competitive inhibitor?*

Due to the weak nature of the FtsYNG:PNA interactions and limited solubility of the PNA, the binding responses in SPR do not go to saturation even at the highest PNA concentrations used. Therefore, the Rmax parameter for the PNA binding was fixed during the curve fitting process and as estimated using the fitted maximum response for the positive control (GDP). We have modified the manuscript text to reflect this and added a reference in Line 477 to our previous paper [1] and ref 19 in manuscript main text.

*6. The RNA-FtsY interaction is well-known to be a transient interaction. Does the PNA have any meaningful inhibitory role in a functional SRP assay (GTP hydrolysis, protein targeting, secretion etc?)*

Given the designed PNA can inhibit the initial step of complex formation according to EMSA, we would expect inhibition of all subsequent functional steps but this remains to be tested. We are planning to carry out more assays to investigate the functions of the PNA on SRP assembly and its biological effects including in vivo activity. However, these are beyond the scope of the current study which provides proof-of-concept that PNAs can be used to specifically target the RNA-binding sites of RBPs in biochemical and biophysical assays.

1.	Faoro C, Wilkinson-White L, Kwan AH, Ataide SF. Discovery of fragments that target key interactions in the signal recognition particle (SRP) as potential leads for a new class of antibiotics. PLoS One. 2018;13(7):e0200387.
